# Public Health Surveillance for Mental Health

**Published:** 2009-12-15

**Authors:** Lisa J. Colpe, Elsie J. Freeman, Tara W. Strine, Satvinder Dhingra, Lisa C. McGuire, Laurie D. Elam-Evans, Geraldine S. Perry

**Affiliations:** Office of Applied Studies, Substance Abuse and Mental Health Services Administration; Maine Department of Health and Human Services, Augusta, Maine; Centers for Disease Control and Prevention, Atlanta, Georgia; Centers for Disease Control and Prevention, Atlanta, Georgia; Centers for Disease Control and Prevention, Atlanta, Georgia; Centers for Disease Control and Prevention, Atlanta, Georgia; Centers for Disease Control and Prevention, Atlanta, Georgia

## Abstract

Public health systems have relied on public health surveillance to plan health programs, and extensive surveillance systems exist for health behaviors and chronic disease. Mental health has used a separate data collection system that emphasizes measurement of disease prevalence and health care use. In recent years, efforts to integrate these systems have included adding chronic disease measures to the Collaborative Psychiatric Epidemiology Surveys and depression measures to the Behavioral Risk Factor Surveillance System; other data collection systems have been similarly enhanced. Ongoing challenges to integration include variations in interview protocols, use of different measures of behavior and disease, different interval reference periods, inclusion of substance abuse disorders, dichotomous vs continuous variables, and approaches to data collection. Future directions can address linking surveillance efforts more closely to the needs of state programs, increasing child health measurements in surveys, and improving knowledge dissemination from survey analyses.

## Introduction

This issue of *Preventing Chronic Disease* addresses the challenges of mental health and mental illness in the public health setting. According to the World Health Organization (WHO), mental illnesses account for more collective disability burden in developed countries than any other group of illnesses, including cancer and heart disease (1). Disability occurs because of both the effect of mental illness on emotions, thoughts, and daily function, and the link between mental illness and general health, especially chronic diseases (2). The Institute of Medicine's (IOM's) Quality Chasm series outlines action steps to improve the quality of all health care, including care for mental health and substance use conditions (2). The IOM notes that failure to provide mental health care also occurs in the United States' public health system: "Despite evidence on risk factors associated with mental illnesses, or the risk factor that mental illness itself may pose to the development of chronic disease, effective public health interventions have not yet been adopted widely in practice" (2).

## Definitions

Public health surveillance is "the ongoing, systematic collection, analysis, interpretation, and dissemination of data regarding a health-related event for use in public health action to reduce morbidity and mortality and to improve health" (3). Historically, surveillance focused on infectious disease, then broadened to other topics, including chronic diseases. Now mental health and mental illness are increasingly recognized as domains in public health surveillance.

Mental health is a state of "successful performance of mental function, resulting in productive activities, fulfilling relationships with other people, and the ability to adapt to change and to cope with adversity" (4). In 2004, WHO published its first report on mental health promotion, conceptualizing mental health as not merely the absence of mental illness but the presence of "a state of well-being in which the individual realizes his or her own abilities, can cope with the normal stresses of life, can work productively and fruitfully, and is able to make a contribution to his or her community" (1). Mental illness has been defined as a separate concept. Mental disorders are characterized by "alterations in thinking, mood, or behavior (or some combination thereof) associated with distress and/or impaired functioning" (4). The remainder of our discussion is focused on mental illness.

## Impact of Mental Illness

An estimated 26% of Americans aged 18 years or older report having a diagnosable mental disorder in a given year (5). The estimated lifetime prevalence of mental disorders among the US adult population is 29% for anxiety disorders, 25% for impulse-control disorders, 21% for mood disorders, 15% for substance use disorders, and 46% for any of these disorders (6). These disorders, especially depression, are among the leading global causes of life years lived with disability (1).

The incidence, course, and outcomes of chronic disease are influenced by mental illness, and the efficacy of interventions for mental illness are affected by the presence of chronic disease. The evidence is extensive for associations between mental illness and medical illnesses such as cardiovascular disease, diabetes, obesity, asthma, epilepsy, and cancer (7,8). The association between mental illness and chronic disease is especially apparent among people with more serious and disabling mental illness, who are at risk of dying 25 years prematurely from cardiovascular and other chronic diseases (9). Research suggests that this association exists not only because of higher rates of smoking and obesity or poor compliance with medical care, but also because of physiologic changes, including endothelial inflammation, platelet stickiness, and changes in the epinephrine-norepinephrine axis and in cortisol metabolism, as well as other metabolites mediated via central nervous system signaling (10).

## Recent Developments in Mental Illness Surveillance

Traditionally, mental health and public health surveillance have operated independent of each other. However, since the release of the first Surgeon General's report on mental health in 1999, government agencies have begun building the infrastructure for establishing an ongoing system for mental health surveillance. Mental health measures are now included in established health surveillance surveys such as the Centers for Disease Control and Prevention's (CDC's) National Health Interview Survey (NHIS), the National Health and Nutrition Examination Survey (NHANES), and the Behavioral Risk Factor Surveillance System (BRFSS); the Agency for Healthcare Research and Quality's Medical Expenditure Panel Survey; the Substance Abuse and Mental Health Services Administration's (SAMHSA's) National Survey on Drug Use and Health (NSDUH); and in the National Science Foundation's Panel Study of Income Dynamics (Table).

Epidemiologic surveys of mental illness, first published by analyzing army recruits in 1942, have been expanded to national populations with the Epidemiologic Catchment Area Study of 1980-1985, the National Comorbidity Survey of 1990-1992, and the National Comorbidity Survey Replication (NCS-R) of 2001-2003 (11). The Collaborative Psychiatric Epidemiology Surveys (CPES) (12) were initiated because of the need for contemporary data about the distributions, social and cultural correlates, and risk factors of mental disorders among the general population and in minority groups. CPES joins together 3 nationally representative surveys: the NCS-R, the National Survey of American Life, and the National Latino and Asian American Study, using an approach that facilitates comparisons across surveys and permits core modules of surveys to be combined into a single data set for analysis. The CPES provides national-level data with sufficient power to investigate cultural and ethnic influences on mental and substance use disorders; mental health service use correlates of interest; and chronic medical conditions such as arthritis, asthma, hypertension, heart disease, headache, allergies, back and neck pain, chronic pain, and diabetes. The data have been used to track the prevalence of mental health conditions for the *Healthy People 2010* objectives and as benchmarks for rates of specific lifetime and previous 12-month mental disorders such as mood, anxiety, and impulse control disorders.

## Measuring the Relationships Between Mental Illnesses, Health Risks, and Chronic Diseases

Understanding the relationship between mental illness and chronic disease is vital to public health assessment and health care delivery. The primary surveillance tool for assessing state-based estimates of health risk behaviors, chronic disease preventive services, and health care access is the BRFSS. The BRFSS is a random-digit–dialed survey of noninstitutionalized people aged 18 years or older, and systems are now in development for expanding its reach. These systems include call-back surveys, special population oversampling, and use of cell phone telephone numbers and paper surveys. BRFSS is unique in that it provides data at the state, county, and metropolitan and micropolitan statistical area levels.

Starting in 2006, state health departments, in collaboration with CDC and the Center for Mental Health Services at SAMHSA, implemented mental illness questions on the BRFSS. From 2006 to 2009, mental health modules have been included in the BRFSS in many states. The Patient Health Questionnaire 8 (PHQ-8), which screens for depressive symptoms, was administered by 41 states and territories in 2006 and 16 states in 2008. The Kessler 6 (K-6) scale, which examines nonspecific psychological distress, was administered in 37 states and territories in 2007 and continues implementation in 2009. Integrated analysis of mental health and other health issues has shown significant associations between mental illness and health risk behaviors (eg, smoking, obesity, physical inactivity), chronic disease (eg, arthritis, diabetes, cardiovascular disease, asthma), and lower levels of preventive care. The BRFSS is among the first surveillance systems that allow population-based state and local estimates for mental and physical health, which are critical for policy and programs at the state and community levels.

Progress has been made with regard to the inclusion of health topics in mental health surveillance at the state level (eg, inclusion of selected BRFSS health questions in the Mental Health Statistics Improvement Program consumer satisfaction survey in several states). This improved surveillance indicates that people with serious mental illness (SMI), defined as having a DSM-IV (*Diagnostic and Statistical Manual of Mental Disorders, Fourth Edition*) mental disorder that resulted in functional impairment, who also have poor physical health, are more likely to have poor functional outcomes and lower levels of satisfaction with their mental health care (13). States can now also analyze their Medicaid services data to examine the effect of co-occurring mental illness, substance use disorders, and chronic medical conditions on utilization and outcomes for chronic disease and behavioral disorders.

Data and instrumentation from the resources listed in the Table are available online for secondary analysis or public use. With the exception of the current (2007 and beyond) NHANES, each of the survey instruments listed contains the K-6 nonspecific psychological distress scale, which is also included in the CPES benchmark studies. This "common currency" approach to instrumentation facilitates general comparisons among studies; however, variations among survey instruments and methods must be considered when interpreting data from different sources.

## Variations Among Surveys

The ways different surveys define and measure mental illnesses often vary. One approach uses interview protocols that ask various symptom, duration, and frequency questions for specific mental disorders, on the basis of classification systems such as the DSM or the International Classification of Diseases (ICD) systems. For example, depression may be measured by using the WHO Composite International Diagnostic Interview (CIDI) depression module, an instrument designed for use in general population surveys, or the PHQ, an instrument designed for use in clinical settings but also used in some health surveys. The WHO CIDI approach provides considerable detail at the national level about specific disorders but requires extensive resources to complete. The PHQ-2, PHQ-8, and PHQ-9, validated measures for depressive symptoms in wide use in clinical care, are sufficiently brief to be included in population-based surveys and public health awareness campaigns.

Another approach is to use different mental health measures to assess "any mental disorder" (as opposed to a specific disorder) by using nonspecific measures of psychological function. These brief scales, commonly found in large, population-based surveys, can be used to identify subpopulations with a high likelihood of having any mental illness. For example, the K-6 scale was originally developed for use in the NHIS as a measure of nonspecific psychological distress (a proxy for poor mental health) during the past 30 days. SAMHSA then supported a pilot study to determine whether a version of the K-6 that assessed distress in the past year could identify SMI cases in the general population (14). In 2008, SAMHSA added impairment and suicide-assessment scales to the NSDUH mental health module and launched a full-scale study to calibrate it to a clinical (psychiatric) evaluation in a nationally representative sample. Results from this study will be used to estimate the prevalence of SMI at the state and national levels.

Another distinction is the reference period, which differs among data collection systems. Serious psychological distress, which is defined as having a K-6 score of 13 or higher during 1 month in the past 12 months, is determined in the NSDUH with the use of a past-year reference period and past-30-day reference period (since 2008). NHIS and BRFSS use a past-30-day reference period only. Different reference periods (current, past year, lifetime) are also found in disorder-specific measures (such as depression), depending on the purpose of the study. The reference period used has a substantial effect on the prevalence rate yielded, not only because the estimates may be subject to recall bias but also because longer reference periods may result in a larger number of affirmative responses.

Other approaches to assessing mental illness have depended on the presence of functional impairments and still others on whether there is a history of treatment. Surveys vary as to the inclusion or exclusion of substance use disorders and cognitive disturbances, such as dementia, under a broader umbrella of mental disorders. These variations in mental illness definitions have provided data on overlapping but nonidentical populations.

Another issue in mental health surveillance is whether specific or nonspecific disorders and associated impairments are measured continuously or dichotomously. Dichotomous measures indicate whether a specific threshold or cutpoint was reached. In contrast, using a continuous measure may allow mild, moderate, and severe levels of disorder to be identified. Studies have shown that untreated minor depression or residual subthreshold depressive symptoms after treatment are often associated with disability and poor psychosocial functioning and a potentially more severe, relapsing, and chronic course that requires additional treatment (15).

The method of data collection may also affect comparability. For example, while most data are collected via computer-assisted interviews, some surveys are self-administered (eg, NSDUH, parts of NHANES are read to respondents via audio-computer technology), and others are administered by an interviewer in the home or on the telephone. These differences affect survey responses, particularly for mental health questions and sensitive items such as illegal or embarrassing behavior (16).

## Future Directions

We have focused on existing efforts in mental illness surveillance but recognize the value of monitoring the prevalence and correlates associated with psychological well-being, as discussed by Manderscheid et al in this issue (17). Future public health surveillance systems should incorporate measures of positive psychological function as both a protective factor against poor health outcomes and a mental health indicator of interest in its own right.

Surveillance efforts can be more meaningfully linked to state and local health policy and program development efforts. Ongoing, multiyear inclusion of mental health modules in state and local level surveys may permit state epidemiologists to monitor the effects of community health partner efforts. We must also develop culturally meaningful surveillance of mental health and mental illness in specific populations, such as ethnic minorities, veterans, parents, caregivers, and people who are disabled.

A lifespan approach to mental health and mental illness will facilitate a better understanding of the natural course of mental illness and its effect on overall health. Most surveys focus on adults and include few measures of child health. Studies of mental health and mental illness in younger populations are relevant to the development of prevention strategies applied at the earlier stages of development.

Evidence-based care is the gold standard for health care providers, but we lack a systematic approach to monitor effectiveness of treatment for mental disorders. Data on medications, unmet needs for mental health services, and outcomes such as employment, housing stability, and consumer satisfaction are available (18,19). However, these measures leave gaps in our understanding of the effect of specific types of treatment in different populations or the relevance of factors such as duration, location, or type of provider. The role of nonprofessional support systems, such as community, spiritual, family, or peer networks, is not fully explored. Effectiveness of treatment has often been measured as if people only have a single mental illness; however, people with these illnesses often have complex comorbidities, including substance abuse, cognitive impairments, and chronic medical conditions. These dimensions should be addressed as well in evaluating efficacy of treatment systems.

Knowledge dissemination is a critical aspect of surveillance, but natural lines of communication in public health and in mental health have generally been separate; information has typically been disseminated from CDC to state and local public health infrastructure and health care providers, and from SAMHSA to state mental health authorities, mental health advocacy organizations, and mental health providers. Systems should be developed so information from an integrated mental health and physical health surveillance system can be directed broadly to specialty mental health and substance abuse providers, public health systems, community health providers, and community health promotion programs.

## Conclusion

In the past decade we have moved from a tradition of separate mental health and public health surveillance efforts to an increasingly integrated approach. Agencies in the US Department of Health and Human Services are collaborating to create new systems and expand existing ones. These systems monitor mental illness by incorporating common mental health measures in ongoing federal surveys, conducting large psychiatric epidemiology benchmark surveys, and facilitating health risk factor surveillance surveys that include mental health variables at the state and local levels. Substantive elements of a mental health surveillance system exist today, although there is work to be done to make an integrated approach standard practice.

Surveillance has focused largely on established disease or symptoms, but collection of additional data on resilience, coping skills, protective factors, and aspects of positive mental health are considerations in devising strategies for disease prevention and mental health promotion. Maintaining focus on the overall health of our population will be critical in the next decades, as will leaving behind the commonly accepted divide between mental and physical illnesses, "despite the fact that both exist within individuals in an exquisitely integrated fashion" (20). An optimal surveillance system will examine interactions among biological, social, psychological, and environmental factors to support health promotion, intervention programs, and both mental illness and chronic disease prevention.

## Figures and Tables

**Table.** United States Surveys That Contain Publicly Available Mental Health Data, 2009

**Survey Type**	Year	Representation	Age Range	Sample Size	Link
**Psychiatric epidemiology studies**					
National Comorbidity Survey Replication	2001-2003	National	≥18 y	9,282	www.hcp.med.harvard.edu/ncs/
National Survey of American Life (black Americans)	2001-2003	National	≥18 y	6,199	www.icpsr.umich.edu/CPES/
National Latino and Asian American Study	2001-2003	National	≥18 y	4,864	www.multiculturalmentalhealth.org/nlaas.asp
**Ongoing health surveys containing mental health questionnaires**					
Behavioral Risk Factor Surveillance System	Ongoing	State	≥18 y	430,000/y	www.cdc.gov/BRFSS/
National Survey on Drug Use and Health	Ongoing	National and state	≥12 y	67,000/y	http://oas.samhsa.gov/nsduh.htm
National Health Interview Survey	Ongoing	National	≥18 y	24,000/y	www.cdc.gov/nchs/nhis.htm
National Health and Nutrition Examination Survey	Ongoing	National	≥12 y	5,000/y	www.cdc.gov/nchs/nhanes.htm
Medical Expenditure Panel Survey	Ongoing	National	All ages	32,000/y	www.meps.ahrq.gov/mepsweb/
Panel Study of Income Dynamics	Ongoing (longitudinal)	National	All ages	7,000 families (total panel)	http://psidonline.isr.umich.edu/Guide/
